# West Nile Virus in Wildlife and Nonequine Domestic Animals, South Africa, 2010–2018

**DOI:** 10.3201/eid2512.190572

**Published:** 2019-12

**Authors:** Jumari Steyn, Elizabeth Botha, Voula I. Stivaktas, Peter Buss, Brianna R. Beechler, Jan G. Myburgh, Johan Steyl, June Williams, Marietjie Venter

**Affiliations:** University of Pretoria, Pretoria, South Africa (J. Steyn, E. Botha, V.I. Stivaktas, J.G. Myburgh, J. Steyl, J. Williams, M. Venter);; South African National Parks, Kruger National Park, South Africa (P. Buss);; Oregon State University, Corvallis, Oregon, USA (B.R. Beechler)

**Keywords:** West Nile virus, viruses, WNV lineage 1, WNV lineage 2, South Africa, wildlife, nonequine domestic animals, zoonoses, vector-borne infections, arboviruses, zoonoses

## Abstract

West Nile virus (WNV) lineage 2 is associated with neurologic disease in horses and humans in South Africa. Surveillance in wildlife and nonequine domestic species during 2010–2018 identified WNV in 11 (1.8%) of 608 animals with severe neurologic and fatal infections, highlighting susceptible hosts and risk for WNV epizootics in Africa.

West Nile virus (WNV) is associated with febrile disease, meningoencephalitis, and death in humans and horses (*1**,**2*). WNV infections are recognized on most continents but remain underreported in Africa. An 8-year study in horses with fever, neurologic signs, or both in South Africa described WNV lineage 2 as the cause of annual outbreaks; 93.7% of WNV-positive horses displayed neurologic signs, resulting in a 34.2% fatality rate (*3*). In the United States, clinical WNV disease has been reported in several nonequine species: birds, crocodiles, bats, wolves, cats, dogs, cattle, and sheep (*4*). The disease susceptibility of wildlife species in Africa and the role they play in amplifying the virus is unknown. We conducted surveillance for neurologic disease and death in animals other than horses in South Africa during 2010–2018 to determine potential WNV reservoir species, identify susceptible hosts, and highlight potential areas for targeted surveillance.

## The Study

A total of 608 specimens comprising central nervous system tissue, visceral organs, and whole blood from wildlife; nonequine domestic animals; and birds with neurologic, febrile, or respiratory signs or sudden unexpected death were submitted to the Centre for Viral Zoonoses, University of Pretoria (Pretoria, South Africa), during February 2010–June 2018. We extracted RNA from the samples using the QIAamp viral RNA (QIAGEN, https://www.qiagen.com) (blood) or RNeasy (QIAGEN) (tissue) mini-kits under Biosafety Level 3 conditions. All specimens were subjected to 1-step nested real-time reverse transcription PCR (RT-PCR) targeting WNV (LightCycler FastStart DNA Master HybProbe; Roche Applied Science, https://www.lifescience.roche.com) (*5*).

Eleven (1.8% [95% CI 0.8%–2.9%]) of the 608 animals tested positive for WNV. A total of 519 (84.5%) specimens were from animals that died, of which 78 were found dead and classified as sudden unexpected death. WNV was detected in 6 (1.7% [95% CI 0.3%–3.0%]) of 361 wildlife and 5 (1.5% [95% CI 0%–3.3%]) of 196 nonequine domestic animals but in 0 of 51 birds (Table 1). We detected WNV RNA in 2 (2%) of 93 domestic cattle (*Bos taurus*), 1 (2%) of 54 African buffalo (*Syncerus caffer*), 1 (5%) of 22 domestic dogs (*Canis lupus familiaris*), 1 (33%) of 3 exotic fallow deer (*Dama dama*), 1 (9%) of 6 giraffes (*Giraffa camelopardalis*), 1 (9%) of 11 domestic goats (*Capra aegagrus hircus*), 1 (11%) of 9 lions (*Panthera leo*), 1 (2%) of 45 domestic sheep (*Ovis aries*), and 2 (7%) of 28 roan antelope (*Hippotragus equinus*) (Table 1). Only 2 of 11 infected animals survived: 1 domestic bovid and the exotic fallow deer.

**Table 1:** West Nile virus detected in specimens from animals with neurologic disease or unexplained death using real-time reverse transcription PCR, South Africa, 2010–2018*

Animal	Identifier	Origin of sample, province	No. positive/animal type (%) [95% CI]	Specimen testing positive	Co-infection (tissue source)
Domestic bovid (*Bos taurus*)	ZRU181/12/1†	Gauteng	2/93 (2.2) [0.0–5.1]	Brain blood	MIDV (spleen)
	ZRU176/14/2	Free State			
African buffalo (*Syncerus caffer*)	ZRU161/18†	Limpopo	1/54 (1.9) [0.0–5.5]	Lung	MIDV (lung, blood)
Domestic dog (*Canis lupus familiaris*)	ZRU358/17†	Gauteng	1/22 (4.6) [0.0–13.3]	Brain, lung	AHSV (brain, lung, spleen)
Fallow deer (*Dama dama*)	ZRU174/14	Gauteng	1/3 (33.3) [0.0–86.7]	Blood	
Giraffe (*Giraffa giraffa*)	ZRU87/18†	North West	1/6 (9.1) [0.0–46.5]	Lung	SHUV (blood)
Domestic goat (*Capra aegagrus hircus*)	ZRU192/14	Gauteng	1/11 (9.1) [0.0–26.1]	Brain, spleen	
Lion (*Panthera leo*)	ZRU297/17†	Mpumalanga	1/9 (11.1) [0.0–31.2]	Brain	
Domestic sheep (*Ovis aries*)	ZRU159/18†	Gauteng	1/45 (2.2) [0.0–6.6]	Spleen	
Roan antelope (*Hippotragus equinus*)	ZRU061/16/2†	Free State	2/28 (7.1) [0.0–16.7]	Lung	
	ZRU165/16	Limpopo		Lung	
Wildlife			6/361 (1.6) [0.3–2.9]		
Domestic animals			5/196 (2.6) [0.3–4.8]		
Birds			0/51		
Total			11/608 (1.8) [0.5–2.9]		

*Identifiers are ZRU laboratory number/year/sample number. AHSV, African horse sickness virus; MIDV, Middleburg virus; SHUV, Shuni virus; ZRU, Zoonosis Research Unit (now CVZ, Centre for Viral Zoonoses). †Sequence data available.

Virus isolation identified African horse sickness virus as a co-infection in the WNV-positive dog (ZRU358_17), confirmed by the Equine Research Centre (*6*) (Table 1). WNV neutralizing antibodies have previously been reported among dogs in South Africa, although no active infection has been described (*7*). The domestic bovid (ZRU181_12_1) and buffalo (ZRU161_18) had Middleburg virus co-infections, and the giraffe had Shuni virus co-infection confirmed by differential testing (*8**–**10*) at the Centre for Viral Zoonoses (Table 1). In these animals, clinical signs and death could not be attributed to any of the detected viruses alone.

**Figure 1:**
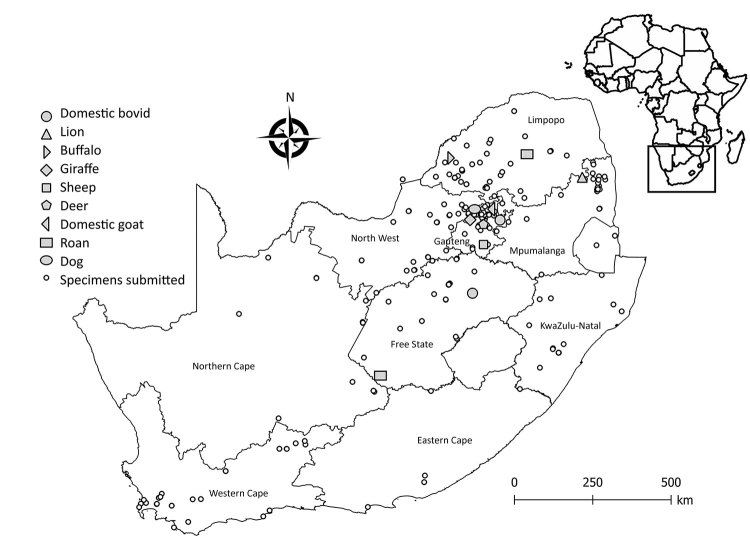
Areas where West Nile virus infections were detected in wildlife and nonequine domestic animals, South Africa, 2010–2018. Insert indicates location of South Africa in Africa.

Positive WNV infections were detected in the Free State (2/45, 4%), Gauteng (5/192, 3%), North West (1/47, 2%), Limpopo (2/132, 2%), and Mpumalanga provinces (1/82, 1%) (Figure 1). Most positive animals were reported during March–June, corresponding to the arbovirus season in South Africa (Appendix).

We detected WNV in lung (5/11, 45%), brain (4/11, 36%), and spleen (2/11, 18%) tissue and in blood (2/11, 18%) (Table 1). Clinical signs noted in WNV-positive animals included neurologic (4/8, 50%) and respiratory (3/8, 38%); 2 animals with neurologic signs also had pyrexia (Table 2). The lion (ZRU297_17) and giraffe (ZRU87_18) were found dead (2/11, 18%); thus, no clinical signs were reported. The WNV-positive sheep (ZRU159_18), an indigenous Dorper, was a stillborn fetus with cerebral edema. In sheep, WNV is reported to cause neurologic symptoms (*11*) but has not been associated with stillbirths. The roan antelope (ZRU61_16_2), the domestic bovid (ZRU181_12_1), and the sheep fetus represented WNV-positive specimens among a cluster of animals with similar signs potentially representing larger outbreaks in these areas. Despite extensive screening for arboviruses, the causative link between the clinical presentation of the various species and the evidence of WNV infection must be regarded with caution because we could not exclude all other possible infectious and noninfectious etiologies.

**Table 2:** Clinical signs and outcomes in wildlife and nonequine domestic animals tested for WNV upon submission to Centre for Viral Zoonoses, South Africa, 2010–2018*

Variable	No. WNV positive/total no. animals (%)	No. WNV negative/total no. animals (%)	Odds ratio (95% CI)	p value†
Sign				
Fever	2/8 (25.0)	44/496 (8.9)	3.4 (0.7–17.2)	0.2
Neurologic signs	4/8 (50.0)	422/496 (85.1)	0.2 (0.0–0.6)	<0.05
Ataxia	2/8 (25.0)	102/496 (20.6)	1.3 (0.3–6.3)	1.0
Paralysis	1/8 (12.5)	63/496 (12.7)	0.9 (0.1–8.0)	1.0
Hind leg paralysis	1/8 (12.5)	22/496 (4.4)	3.0 (0.4–25.7)	0.3
Paresis	2/8 (25.0)	118/496 (23.8)	1.1 (0.2–5.3)	1.0
Tongue paralysis	0/8	4/496 (0.8)	Undefined	1
Recumbency	2/8 (25.0)	103/496 (20.8)	1.3 (0.3–6.3)	0.7
Dyspnea	3/8 (37.5)	78/496 (15.7)	3.2 (0.7–13.5)	0.1
Hemorrhage	0/8	11/496 (2.2)	Undefined	1
Blindness	0/8	11/496 (2.2)	Undefined	1
Icterus	0/8	2/496 (0.4)	Undefined	1
Seizure	0/8	30/496 (6.0)	Undefined	1
Outcome‡				
Sudden unexpected death	2/11 (18.2)	76/608 (12.5)	1.5 (0.3–7.2)	0.4
Stillborn	1/11 (9.1)	15/608 (2.5)	3.9 (0.5–32.3)	0.3
Abortion	0/11	24/608 (4.0)	Undefined	1
Congenital deformities	0/11	11/608 (1.8)	Undefined	1
Death	9/11 (81.8)	510/608 (84.4)	0.8 (0.2–3.6)	0.4

*WNV, West Nile virus. †p values <0.05 are significant. ‡Sudden unexplained death indicates animals found dead without an obvious reason; stillborn, abortion, and congenital deformities are related to potential cross-placental transmission; death refers to sick animals that subsequently died.

**Figure 2:**
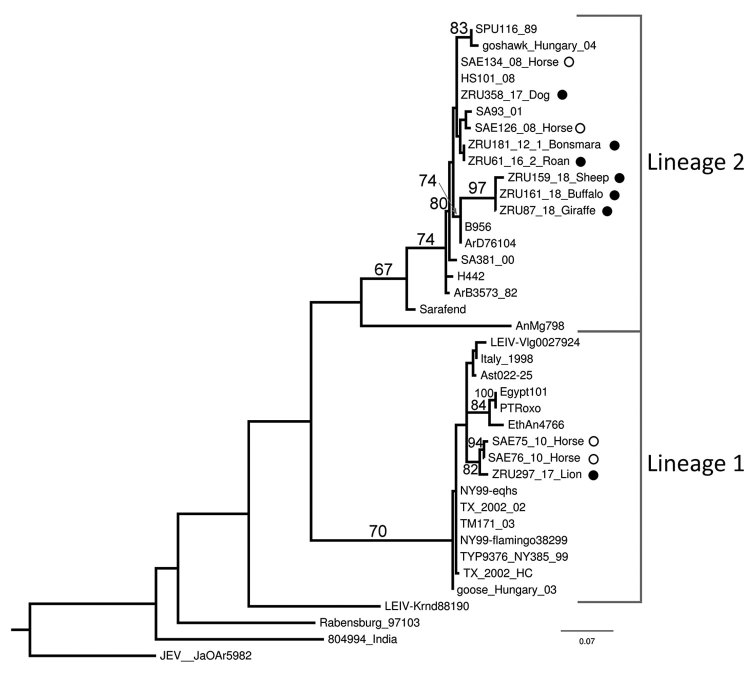
Maximum-likelihood phylogram of the partial (215-nt) nonstructural protein gene used for identification of West Nile virus infection in wildlife and nonequine domestic animals, South Africa, 2010–2018. Tree was generated with RAXML (https://cme.h-its.org/exelixis/web/software/raxml) using the general time-reversible plus gamma model with 39 taxa and the AutoMRE bootstopping function invoked (bootstraps >65 as branch support). Black circles indicate wildlife and nonequine domestic animal sequences from this study; open circles indicate horse sequences (*3**,**12*). Reference strains, GenBank accession numbers, and origins are as indicated in (*4*). GenBank accession numbers for the newly sequenced strains are ZRU87_18, MN270988; ZRU159_18_SA, MN270989; ZRU161_18_SA, MN27099; and ZRU181_12_1, KY176733. The sequences for strains ZRU358/17, ZRU061/16/2, and ZRU297/17 were <200 bp long and therefore could not be submitted to GenBank; the sequence data are available from the authors. Scale bar indicates nucleotide substitutions per site.

We subjected positive specimens to Sanger sequencing (Inqaba biotech, https://www.inqababiotec.co.za) and conducted sequence analysis with CLC-genomic workbench (https://www.qiagenbioinformatics.com), MAFFT (Multiple Alignment using Fast Fourier Transform) version 7 (http://mafft.cbrc.jp/alignment/server), and MEGA6.06 (https://www.megasoftware.net). We used RAxML (https://cme.h-its.org/exelixis/web/software/raxml) for maximum-likelihood phylogenetic analysis of the partial nonstructural protein 5 gene region (215 nt) and confirmed the RT-PCR results and WNV lineages (Figure 2). The lion from Kruger National Park (KNP) clustered with lineage 1 (bootstrap = 70), and all other animals clustered with lineage 2 strains from South Africa (bootstrap = 67) (Figure 2). One previous report found a lineage 1 strain that clustered with lineage 1 strains previously identified in South Africa (*12*).

We used an epitope-blocking ELISA (*13*) to screen serum for WNV antibodies in 50 white rhinoceros (*Ceratotherium simum*) collected by the South African National Parks in March 2014 and 45 African buffalo in June 2016, all from KNP, and from 34 Nile crocodiles (*Crocodylus niloticus*) collected from northern KwaZulu-Natal during 2009–2012. We coated flat-bottom 96-well microtiter plates (CELLSTAR, Sigma Aldricht, https://www.sigmaaldrich.com) with 1:800 dilution of WNV cell lysate antigen, prepared according to (*14*) using strain HS101/08, passage 6, South Africa and WNV hyperimmune mouse ascites fluid polyclonal antibody (FC-M30200-06-1, Centers for Disease Control and Prevention, https://www.cdc.gov/ncezid/dvbd/specimensub/arc) diluted 1:400 and horseradish peroxidase–conjugated rabbit antimouse IgG (BioRad Laboratories, https://www.bio-rad.com) (1:2000 dilution). We calculated the percentage inhibition of antibody binding with a cutoff value of 40% and confirmed positive reactions by microtiter virus neutralization test using a 10^3^ 50% tissue culture infectious dose stock culture (MRM61C, passage 6) (*15*). We detected WNV-specific antibodies in serum of 25 (50%) of white rhinoceros, of which 20 (80%) demonstrated neutralization at all 3 dilutions (1:8, 1:16, and 1:32) and 5 showed no neutralization, suggesting high-level WNV exposure. This finding highlights the prevalence of WNV in KNP despite a low number of reported clinical infections. No buffaloes or crocodiles were seropositive.

## Conclusions

We recorded WNV (lineages 1 and 2) in wildlife and nonequine domestic animals in South Africa. Seroconversion to WNV was demonstrated in asymptomatic white rhinoceros from KNP. The data suggest severe disease and neurologic signs occur in species other than horses; these signs may be used for surveillance in areas of Africa where horses are less common to predict WNV outbreaks and predict spillover events into the human population. Wildlife and nonequine domestic animals are not as closely monitored for WNV as horses, and early detection is less likely. The short viremia associated with WNV infection may result in underreporting of positive animals if only RT-PCR is used for diagnosis, but a lack of conjugates for wildlife species complicates development of IgM ELISA. The epitope-blocking ELISA and microtiter virus neutralization test can be used for seroprevalence studies in animals other than horses because they are species-independent but do not differentiate between IgM and IgG and are not quantitative. Future work should focus on assay development for species other than horses.

Appendix Seasonality of West Nile virus in wildlife, nonequine domestic animals, and birds, South Africa, 2010–2018.
