# Supplementary material for: West Nile Virus in Wildlife and Nonequine Domestic Animals, South Africa, 2010–2018
Source: Emerg Infect Dis. 2019 Dec;25(12):2290–4. doi: 10.3201/eid2512.190572 (PMC6874268; doi:10.3201/eid2512.190572)
Supplement: Appendix — Seasonality of West Nile virus in wildlife, nonequine domestic animals, and birds, South Africa, 2010–2018. [file 19-0572-Techapp-s1.pdf]

Article DOI: https://doi.org/10.3201/eid2512.190572

West Nile Virus in Wildlife and Nonequine Domestic Animals, South Africa, 2010–2018

Appendix

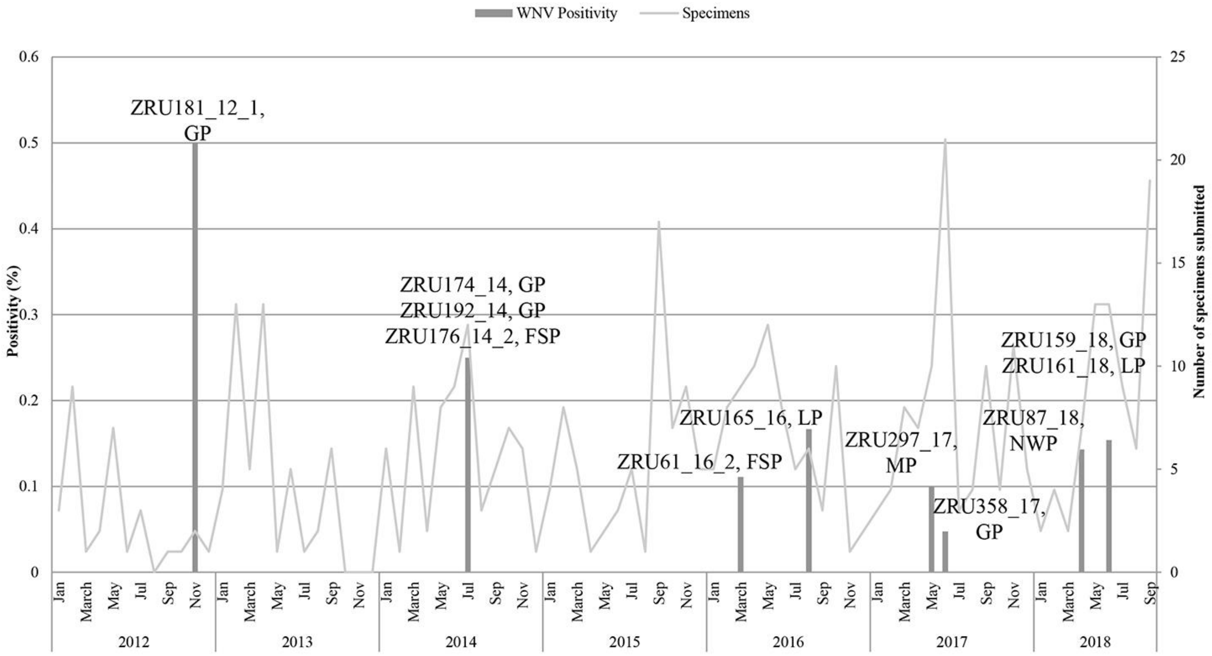

Appendix Figure. Seasonality of wildlife, nonequine domestic animals, and avian specimens (N = 608) submitted and West Nile virus reverse transcription PCR–positives (n = 11), South Africa, 2012–2018. West Nile virus PCR-positive results are indicated in the graph. FSP, Free State province; GP, Gauteng province; LP, Limpopo province; MP, Mpumalanga province; NWP, North West province.
